# A retrospective study: the prevalence and prognostic value of anemia in patients undergoing radiotherapy for esophageal squamous cell carcinoma

**DOI:** 10.1186/1477-7819-12-244

**Published:** 2014-08-01

**Authors:** Fang Zhang, Fengyu Cheng, Lifang Cao, Shengchuan Wang, Wei Zhou, Wei Ma

**Affiliations:** 1 Cancer Center, the First Hospital of Zibo, 4 East Emei mountain Rd, Zibo 255200, Shandong, China; 2 Department of Radiotherapy, Cancer Centre, Qilu Hospital of Shandong University, 107 West Wenhua Rd, Jinan 250012, Shandong, China; 3 Cancer Centre, Liaocheng People’s Hospital, Liaocheng 252000, Shandong, China

**Keywords:** Anemia, Survival, Prevalence, Esophageal neoplasms, Squamous cell carcinoma, Radiotherapy

## Abstract

**Background:**

The relationship between anemia and outcomes after radiotherapy has not been systematically addressed. The study aimed to assess the prevalence and prognostic value of anemia in patients receiving primary radiotherapy for esophageal squamous cell carcinoma (ESCC).

**Methods:**

A total of 103 patients with ESCC were retrospectively reviewed. Anemia was defined as a hemoglobin level <12 g/dl for men and <11 g/dl for women. The 3-year and 5-year overall survival (OS) and disease-free survival (DFS) were analyzed between the anemic and non-anemic groups using the Kaplan-Meier method and the Cox proportional hazards model.

**Results:**

No significant differences were observed in patient characteristics between the anemic and non-anemic groups. The prevalence of anemia was 29.1%. The 3-year and the 5-year OS were 43% and 37%, respectively, in the non-anemic group, and 20% and 17%, respectively, in the anemic group. The 3-year and the 5-year DFS were 37% and 26%, respectively, in the non-anemic group, and 13% and 10%, respectively, in the anemic group. Survival analysis using the Kaplan-Meier method showed that there was significant difference between anemia and non-anemia (*P* < 0.02). In a multivariate analysis, anemia was identified as a highly significant prognostic factor for 3-year OS (hazard ratio 1.916; *P* = 0.012) and 3-year DFS (hazard ratio 1.973; *P* = 0.007), independent of T stage and the status of lymph nodes, and 5-year OS (hazard ratio 1.705; *P* = 0.027) and 5-year DFS (hazard ratio 1.980; *P* = 0.005), independent of TNM stage and the status of lymph nodes.

**Conclusions:**

Anemia before primary radiotherapy was associated with poor prognosis and an increased risk of relapse, which may serve as a new prognostic factor for ESCC.

## Background

Esophageal carcinoma is the eighth most common cancer and the sixth highest cancer risk for mortality in the world [1]. There is a remarkable geographic variation in esophageal cancer incidence [2]. China has a high incidence of esophageal cancer, about 20 times higher than that in low-incidence areas of Africa [3,4]. There are two major histological types of esophageal carcinoma: esophageal squamous cell carcinoma (ESCC) and adenocarcinoma. ESCC continues to be the major type of esophageal cancer in China and other East Asian countries, whereas adenocarcinoma is more common in the United States and European countries.

Anemia is known to be a common condition in cancer patients, and about 30% of cancer patients suffer from anemia [5,6]. Disorders of iron metabolism, blood marrow insufficiency or metastases, malnutrition, bleeding at tumor site, catabolism of patients with tumor burden and relative deficiency of erythropoietin all play a role in anemic pathogenesis. Anemia was found to be an independent prognostic factor for poor survival in solid malignant tumors and hematologic malignancies in a meta-analysis [5]. Anemia is an indicator of poor prognosis in T1-T2 squamous cell carcinoma of the glottic larynx [7]. In a series of 217 patients with squamous cell carcinomas in the head and neck treated with curative radiation therapy alone, Dubray and colleagues [8] found that the 2-year local-regional control rate decreased and the relative risk of death increased for anemia. Grigiene and colleagues [9] analyzed the outcome of 162 patients with uterine cervical carcinoma treated with irradiation and found that the hemoglobin (Hb) level before treatment had a significant influence on overall survival (OS), disease-free survival (DFS) and local relapse-free survival.

However, little is known about the significance of anemia in the outcome of ESCC patients undergoing primary radiotherapy. The aim of this study was to evaluate the prevalence and prognostic value of anemia in ESCC and its relationship with other prognostic factors.

## Methods

### Patients

Between 1 January 2006 and 31 December 2007, 154 patients who underwent primary radiotherapy at the Department of Radiation Oncology, Qilu Hospital of Shandong University, Jinan, China, were enrolled in this study. The exclusion criteria were patients with the non-squamous cell subtype, patients without record of Hb, and patients who died of complications of radiotherapy. Consequently, 103 patients were available for the present study.

All non-surgical patients in this study were staged according to routine practice with air contrast barium esophagography, upper gastrointestinal endoscopy with histological biopsies and cervical, chest and abdominal contrast computed tomography. All surgical patients were staged in accordance with the American Joint Committee on Cancer TNM staging system [10]. All patients received radiotherapy alone or post-operative radiotherapy or radiochemotherapy according to the practice. Radiotherapy was started on day 1 and delivered at 2 Gy/day for 5 days a week for a total radiation dose of 60-70 Gy for non-surgical patients, and a total radiation dose of 50 Gy for surgical patients.

### Definition of anemia

The definition of anemia used in this study was kept consistent with the definitions used by our laboratory and China-specific criteria: a Hb level of less than 12 g/dl for men and less than 11 g/dl for women [11]. A patient was classified as being anemic if their mean Hb met the criteria before radiotherapy.

### Follow-up

Follow-up data were collected until death or 31 December 2012. All patients had a regular follow-up schedule including a complete history and physical examination every 3 months during the first 2 years, every 6 months during the first 3 to 5 years and every year thereafter. Routine radiological examinations were performed when necessary.

### Statistical analysis

Differences in patient characteristics were assessed using the Mann–Whitney test for continuous variables and the χ^2^ test for categorical variables. Analysis and comparison of survival curves were performed using Kaplan–Meier curves and log-rank analysis. For the analysis of 3-year and 5-year OS, events were defined as death from any cause. For the analysis of 3-year and 5-year DFS, events were defined as first loco-regional or distant tumor relapse or death from any cause. The Cox proportional hazards model was used to determine the hazard ratio (HR) of variables on 3-year and 5-year OS and DFS in univariate and multivariate analysis. The results were given as HR with their 95% CI. All statistical tests were two-sided and *P* < 0.05 was considered as significant. Data were analyzed using statistical package SPSS version 17.0 (SPSS Inc., Chicago, IL, USA).

## Results

### Characteristics of the patients

A total of 103 patients (91 men and 12 women) were retrospectively analyzed in this study. The median age of the patients was 60 years (range 41 to 83 years) at the date diagnosed. The median follow-up time was 18 months (range 3 to 87 months). There were no significant differences between non-anemic group and anemic group in age (*P* = 0.796), gender (*P* = 0.738), tumor location (*P* = 0.548), treatment modality (*P* = 0.519), TNM stage (*P* = 0.296), T stage (*P* = 0.201) and the status of lymph nodes (*P* = 0.357). Patient characteristics are presented in Table 1.

**Table 1:** Baseline and treatment variables in anemic and non-anemic patient groups

		**Anemic**	**Non-anemic**	***P *** **value**
Age (years)		61 (41-82)	60 (45-83)	0.796
Male:female		27:3	64:9	0.738
Hemoglobin (g/dl)	Male	11.4 (8.6-11.9)	13.95 (12.0-15.8)	0.000
	Female	10.3 (8.1-10.8)	13.1 (11.7-14.6)	0.000
Tumor location	Cervical	5	8	0.548
	Upper	7	14	
	Middle	11	38	
	Low	7	13	
Treatment modality	Radiotherapy	11	21	0.519
	Surgery + radiotherapy	9	31	
	Chemoradiotherapy	5	7	
	Surgery + Chemoradiotherapy	5	14	
TNM stage	I	3	4	0.296
	II	13	31	
	III	11	36	
	IV	3	2	
T stage	T1	3	8	0.155
	T2	9	13	
	T3	17	38	
	T4	1	14	
Status of lymph nodes	Negative	11	34	0.390
	Positive	19	39	
Values are shown as median (range) or number, as appropriate.				

### The prevalence of anemia

Anemia was diagnosed in 27 (29.7%) men and three (25%) women. There were 30 anemic patients in 103 patients. The median Hb levels were 13.9 g/dl in the non-anemic group and 11.3 g/dl in the anemic group. The prevalence of anemia in ESCC patients undergoing radiotherapy was 29.1%.

### Correlation of anemia with patient survival

**Figure 1:**
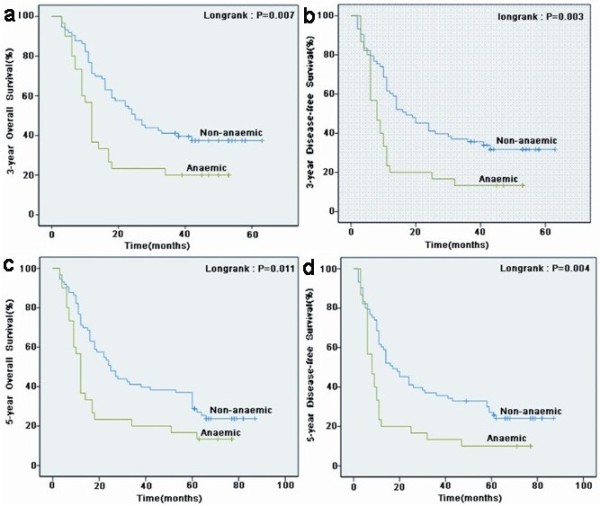
**Kaplan-Meier curves for the survival of all 103 patients with esophageal squamous cell carcinoma.** Both 3-year overall survival **(a)** and disease-free survival **(b)** between the non-anemic and anemic groups were statistically significant. Both 5-year overall survival **(c)** and disease-free survival **(d)** between the non-anemic and anemic groups were statistically significant.

The 3-year and 5-year OS were 43% and 37%, respectively, in the non-anemic group, and were 20% and 17%, respectively, in the anemic group. The 3-year and the 5-year DFS were 37% and 26%, respectively, in the non-anemic group, and 13% and 10%, respectively, in the anemic group. The Kaplan-Meier method showed that both 3-year and 5-year OS and DFS in the non-anemic group were significantly better than those in the anemic group (*P* < 0.05; Figure 1).

The Cox univariate model showed that anemia, the status of lymph node, TNM stage and T stage were significantly correlated to 3-year and 5-year OS and DFS. The HRs for 3-year and 5-year OS with anemia were 1.941 (95% CI 1.177 to 3.202; *P* = 0.009) and 1.799 (95% CI 1.123 to 2.882; *P* = 0.015), respectively. The HRs for 3-year and 5-year DFS with anemia were 1.999 (95% CI 1.232 to 3.244; *P* = 0.005) and 1.946 (95% CI 1.217 to 3.112; *P* = 0.005), respectively. Anemia before radiotherapy was associated with poor prognosis and an increased risk of relapse (Tables 2 and 3).

**Table 2:** Cox univariate analysis for 3-year survival in 103 patients

	**3-Year overall survival**			**3-Year disease-free survival**		
	**HR**	**95% CI**	***P***	**HR**	**95% CI**	***P***
Hemoglobin (non-anemic vs anemic)	1.941	1.177-3.202	0.009	1.999	1.232-3.244	0.005
Tumor location (C vs U vs M vs L)	0.936	0.720-1.218	0.623	0.883	0.686-1.136	0.333
Treatment modality (RT vs S + RT vs CRT vs S + CRT)	0.795	0.615-1.027	0.079	0.874	0.697-1.096	0.244
TNM stage (I vs II vs III vs IV)	1.767	1.240-2.519	0.002	2.052	1.431-2.945	0.000
T stage (T1 vs T2 vs T3 vs T4)	1.409	1.017-1.952	0.039	1.543	1.125-2.116	0.007
Status of lymph node (negative vs positive)	2.676	1.582-4.527	0.000	2.241	1.376-3.652	0.001

C, cervical; CRT, chemoradiotherapy; HR, hazard ratio; L, low; M, middle; RT, radiotherapy; S, surgery; U, upper.

**Table 3:** Cox univariate analysis for 5-year survival in 103 patients

	**5-Year overall survival**			**5-Year disease-free survival**		
	**HR**	**95% CI**	***P***	**HR**	**95% CI**	***P***
Hemoglobin (non-anemic vs anemic)	1.799	1.123-2.882	0.015	1.946	1.217-3.112	0.005
Tumor location (C vs U vs M vs L)	0.945	0.739-1.208	0.650	0.935	0.729-1.199	0.595
Treatment modality (RT vs S + RT vs CRT vs S + CRT)	0.856	0.681-1.076	0.183	0.916	0.741-1.133	0.420
TNM stage (I vs II vs III vs IV)	1.732	1.240-2.418	0.001	1.783	1.263-2.518	0.001
T stage (T1 vs T2 vs T3 vs T4)	1.319	0.988-1.761	0.060	1.441	1.071-1.939	0.016
Status of lymph node (negative vs positive)	2.216	1.394-3.523	0.001	1.769	1.127-2.776	0.013

C, cervical; CRT, chemoradiotherapy; HR, hazard ratio; L, low; M, middle; RT, radiotherapy; S, surgery; U, upper.

Using multivariate analysis, anemia was identified as a significant prognostic factor for 3-year OS (HR 1.916; *P* = 0.012) and 3-year DFS (HR 1.973; *P* = 0.007), independent of T stage and the status of lymph nodes, and for 5-year OS (HR 1.705; *P* = 0.027) and 5-year DFS (HR 1.980; *P* = 0.005), independent of TNM stage and the status of lymph nodes (Table 4 and 5).

**Table 4:** Cox multivariate analysis for 3-year survival in 103 patients

	**3-Year overall survival**			**3-Year disease-free survival**		
	**HR**	**95% CI**	***P***	**HR**	**95% CI**	***P***
Hemoglobin (non-anemic vs anemic )	1.916	1.155-3.180	0.012	1.973	1.204-3.233	0.007
T stage (T1 vs T2 vs T3 vs T4)	1.390	0.980-1.971	0.065	1.607	1.144-2.258	0.006
Status of lymph node (negative vs positive)	2.456	1.448-4.165	0.001	1.994	1.218-3.263	0.006

**Table 5:** Cox multivariate analysis for 5-year survival in 103 patients

	**5-Year overall survival**			**5-Year disease-free survival**		
	**HR**	**95% CI**	***P***	**HR**	**95% CI**	***P***
Hemoglobin (non-anemic vs anemic )	1.705	1.063-2.737	0.027	1.980	1.232-3.180	0.005
TNM stage (I vs II vs III vs IV)	1.428	0.986-2.069	0.060	1.771	1.265-2.479	0.001
Status of lymph node (negative vs positive)	1.738	1.051-2.875	0.031	1.304	0.724-2.347	0.377

## Discussion

In the present study, the prevalence of anemia in patients with ESCC undergoing primary radiotherapy was 29.1%, consistent with the previously reported prevalence of anemia in squamous cell carcinomas of the head and neck (29%) [8]. Similarly, the prevalence of anemia was 35% in rectal carcinoma patients undergoing neoadjuvant radiotherapy, and 44% in advanced Ewing’s sarcoma patients undergoing chemotherapy [12,13].

Recent studies showed that anemia was associated with poor prognosis and an increased risk of relapse [14,15]. In our recent study, we found that anemia was identified as a highly significant prognostic factor for 2-year OS and DFS, independent of TNM stage and initial treatment in ESCC treated with primary radiotherapy [16]. In order to investigate whether anemia would continue to affect survival, we analyzed patients between 1 January 2006 and 31 December 2007 and concluded similar results. In our current study, we found that 3-year and 5-year OS were 43% and 37%, respectively, in the non-anemic group, and 20% and 17%, respectively, in the anemic group. Moreover, 3-year and 5-year DFS were 37% and 26%, respectively, in the non-anemic group, and 13% and 10%, respectively, in the anemic group. Both 3-year and 5-year OS and DFS in the non-anemic group were significantly better than those in the anemic group (*P* < 0.05). Similarly, Zhao and colleagues [17] analyzed the effect of anemia on survival in 303 patients with locally advanced esophageal carcinoma undergoing irradiation and reported that there was a statistically significant reduction in survival and loco-regional control rates.

Several hypotheses have been proposed to explain the relationship between anemia and cancer [18-21]. First, anemia is known to produce tumor hypoxia which has oncogenic actions through the associated genomic instability, mutagenesis and disordered cell growth [18,19]. Tumor hypoxia also confers radio-resistance through the hypoxia-associated reduction in free-radical production and consequent radiotherapy-induced DNA damage [20]. Moreover, tumor hypoxia is associated with a poor prognosis in patients with advanced head and neck cancer [21]. Second, bulky tumors might have a more anemic condition compared with small volume tumors. In patients with early cervical cancer, the prevalence of pretreatment anemia was 12.3% which is a relatively lower incidence than that of locally advanced cervical cancer reported in previous studies (approximately 25%) [22-24]. Moreover, a review article also reported that Hb levels prior to and during radiotherapy in the patients with cervical cancer are strongly correlated with tumor size (*P* < 0.001) [25]. It may partially explain the prognostic impact on survival in patients with cervical cancer. Third, Barkati and colleagues [26] reported that a lower Hb level was related to a more infiltrative and aggressive disease, such as uterine corpus invasion and nodal metastases, in 263 patients with locally advanced cervical cancer.

In the present study, the prevalence of anemia was 31.4% in I and II stage patients, and 26.9% in III and IV stage patients. The prevalence of anemia was 36.4% in T1 and T2 stage patients, and 25.7% in T3 and T4 stage patients. The results did not show that the patients with stages III and IV, and T3 and T4 might have a more anemic condition than the patients with stages I and II, and T1 and T2; however, the proportion of anemia in patients without lymph nodes metastases was 24.4%, which is a relatively lower incidence than that of patients with lymph nodes metastases ( 32.8%). The results suggested that patients with lymph nodes metastases might have a more anemic condition compared with patients without lymph nodes metastases, and this is one possible explanation for poor survival and anemia in ESCC. Overall, it is clear that the anemic group has an inferior prognosis in the study, but the underlying mechanisms remain unknown. Further research is needed to detect the mechanisms.

Many prognostic factors have been found for esophageal cancer, such as the status of lymph nodes, TNM stage, tumor location, and T stage. All these factors have been included in our analysis. In this study, we found that the status of lymph node, TNM stage and T stage were significantly correlated to 3-year OS and 3-year DFS by univariate analysis. Using multivariate analysis, the status of lymph node was an independent prognostic factor for 3-year OS, while the status of lymph node and T stage were independent prognostic factors for 3-year DFS. Furthermore, we found that the status of lymph node and T stage were significantly correlated to 5-year OS by univariate analysis, and the status of lymph node was an independent prognostic factor by multivariate analysis. For 5-year DFS, the status of lymph node, TNM stage and T stage were independent prognostic factors by univariate analysis, and TNM stage was an independent prognostic factor by multivariate analysis. Our results are similar to those of previous studies [17,27,28].

In present study there are several limitations. First, we did not account for the possible correction of anemia before primary radiotherapy; blood transfusion and administration of erythropoietin in cancer patients may help improve anemia and patient survival [29]. Second, treatments such as surgery and chemotherapy partially contribute to the anemic condition before radiotherapy in patients with ESCC. Third, anemia before primary radiotherapy could be frequently associated with symptoms such as malnutrition and bleeding at the tumor site as the blood test was performed. As ours was a retrospective study, we could not consider these confounding factors or the comorbid conditions of the patients, which may affect our results. Hence, it is difficult to judge anemia as a direct or indirect prognostic factor for poor survival; however, it is clear that the anemic group has a shorter survival. Further research with larger sample sizes is needed to validate these findings.

## Conclusion

Our findings indicate that anemia before primary radiotherapy is a new prognostic factor for ESCC, independent of other prognostic factors. Hb is a routine examination and thus anemia can be easily assessed to provide a prediction for the prognosis of ESCC patients.

## Consent

Written informed consent was obtained from the patients for publication of this research article and any accompanying images.

## Abbreviations

DFS: disease-free survival; ESCC: esophageal squamous cell carcinoma; Hb: hemoglobin; HR: hazard ratio; OS: overall survival.

## Competing interests

The authors declare that they have no competing interests.

## Authors’ contributions

ZF conceived the study, participated in its design, performed the statistical analysis and drafted the manuscript. FC participated in the design of the study and coordination, and helped to draft the manuscript. LC and SW participated in the analysis of experimental results. WZ and WM followed-up all patients. All authors read and approved the final manuscript.
